# Cognitive Dysfunction, Affective States, and Vulnerability to Nicotine Addiction: A Multifactorial Perspective

**DOI:** 10.3389/fpsyt.2016.00160

**Published:** 2016-09-21

**Authors:** Morgane Besson, Benoît Forget

**Affiliations:** ^1^Unité de Neurobiologie Intégrative des Systèmes Cholinergiques, Department of Neuroscience, CNRS UMR 3571, Institut Pasteur, Paris, France

**Keywords:** nicotine, predisposition, psychiatric disorders, cognition, addiction, emotion

## Abstract

Although smoking prevalence has declined in recent years, certain subpopulations continue to smoke at disproportionately high rates and show resistance to cessation treatments. Individuals showing cognitive and affective impairments, including emotional distress and deficits in attention, memory, and inhibitory control, particularly in the context of psychiatric conditions, such as attention-deficit hyperactivity disorder, schizophrenia, and mood disorders, are at higher risk for tobacco addiction. Nicotine has been shown to improve cognitive and emotional processing in some conditions, including during tobacco abstinence. Self-medication of cognitive deficits or negative affect has been proposed to underlie high rates of tobacco smoking among people with psychiatric disorders. However, pre-existing cognitive and mood disorders may also influence the development and maintenance of nicotine dependence, by biasing nicotine-induced alterations in information processing and associative learning, decision-making, and inhibitory control. Here, we discuss the potential forms of contribution of cognitive and affective deficits to nicotine addiction-related processes, by reviewing major clinical and preclinical studies investigating either the procognitive and therapeutic action of nicotine or the putative primary role of cognitive and emotional impairments in addiction-like features.

## Introduction

Smoking tobacco remains the most preventable cause of morbidity and mortality worldwide. Nicotine is the main psychoactive component of tobacco responsible for its addictive properties and modifies the function of the brain *via* its interaction with the nicotinic acetylcholine receptors (nAChRs) (1, 2). Drug addiction is a complex psychiatric disorder, and there are individual differences in the vulnerability to develop this pathology that can be conceptualized at different levels interacting with each other, such as environmental, genetic, and psychological contributions. Only a percentage of individuals starting to smoke tobacco eventually develop an addiction (3). In particular, there is a high prevalence of smoking in patients with psychiatric disorders. However, it has been difficult to define in clinical studies the nature of the causal interactions between these pathologies. The psychological and neural processes that underlie addiction have been shown to overlap with those that support cognitive and emotional functions. One critical question is to which extent psychiatric conditions may pre-date smoking or develop after chronic exposure to nicotine. One of the main limitations to resolve this issue is the difficulty to conduct longitudinal prospective studies in humans and to control for co-use of multiple substances in patient cohorts. As a consequence, preclinical research has increasingly aimed at identifying distinctive endophenotypes that may predispose individuals to nicotine addiction-like processes and/or that are influenced by nicotine exposure. Animal models can never encompass entirely the complexity of the psychological processes underlying behavior related to addiction and other psychiatric conditions in humans with full face and construct validities. Yet, they provide a valuable tool to precisely control the environmental (and genetic) context, the conditions of drug delivery, and to determine whether beforehand drug consumption influences the risk to develop specific endophenotypes or whether pre-existing endophenotypes confer vulnerability to addiction, through the implementation of longitudinal studies. They also allow detailed investigations of the distinct stages of addiction that may be connected to some endophenotypes to varying extents. In fact, the defining criteria of addiction are still a matter of debate, and this pathology exhibits complex dynamics with different stages, from the initiation and maintenance of drug taking to a switch toward a loss of control over drug intake, compulsive drug taking and seeking, i.e., despite negative consequences, together with high rate of relapse after abstinence (4–8). With the use of experimental models of distinct addiction-like behaviors in addition to epidemiological and neurocognitive studies in human subjects, specific behavioral endophenotypes of presumed genetic origin have been identified as significant risk factors for drug addiction according to different modalities. Understanding the causal relationship between nicotine addiction and psychiatric disorders may significantly contribute to the treatment of comorbid psychiatric conditions and smoking. This review will describe and discuss both clinical and preclinical studies that brought significant insight in that matter.

## Tobacco Smoking, Personality Traits, and Psychiatric Conditions

Vulnerability to addiction varies across individuals. Thus, although many people experiment with drugs of abuse, most do not develop drug addiction as defined by diagnostic criteria for substance-use disorder (9). Individual differences in vulnerability to abuse are thought to exist before the first drug experience and clinical evidence suggests that these differences reflect both genetic and environmental determinants, including social influences, as well as their interaction [see Ref. (10) for review]. Cigarette smoking is the leading preventable cause of death in the Western world (11) with a prevalence considerably higher in individuals with psychiatric diagnosis. In this part of the review, we will examine non-exhaustively the relationships described in clinical studies between smoking behavior, personality traits, and psychiatric disorders, such as impulsivity, novelty/sensation seeking, attention-deficit hyperactivity disorder (ADHD), depression, and anxiety disorders (see Table 1).

**Table 1:** **Mental disorders/personality trait and nicotine addiction-related features in humans**.

Mental disorder/personality trait	Nicotine addiction-related features	Reference
Cognitive impulsivity	↗ initiation of smoking behavior in adolescents	**Audrain-McGovern et al. (12)**
	↗ smoking relapse	Sheffer et al. (13)
Motor impulsivity	↗ subjective rewarding effects of nicotine	Perkins et al. (14)
	↗ risk for regular tobacco smoking	**Anokhin and Golosheykin (15)**
Impulsivity (subtype undetermined)	↗ explicit expectancies about nicotine reward	Doran et al. (16)
	↗ initiation of smoking	**Lipkus et al. (17)**
Novelty/sensation seeking	↗ risk to become regular smoker	**Audrain-McGovern et al. (18)**
	↗ sensitivity to the initial reinforcing effect of nicotine	Perkins et al. (14)
	↗ initiation of smoking	**Lipkus et al. (17)**
	↘ smoking-cessation success	Kahler et al. (19), Batra et al. (20)
ADHD	↗ future smoking	Fuemmeler et al. (21), **Tercyak et al. (22)**
	↗ relapse to smoking	Humfleet et al. (23)
	↘ onset of regular smoking	**Lambert and Hartsough (24)**, **Kollins et al. (25)**
	↗ withdrawal symptoms	Pomerleau et al. (26), McClernon et al. (27), Kollins et al. (28)
	↗ motivation for cigarette puffs	Kollins et al. (28)
	↗ nicotine dependence	**Wilens et al. (29)**
Major depression	↗ smoking and risk of nicotine dependence	**Fergusson et al. (30)**
	↘ likeliness to quit	**Rohde et al. (31)**
	↘ odds of smoking abstinence	Glassman et al. (32), Hitsman et al. (33)
Depression symptoms	↗ smoking initiation	**Escobedo et al. (34)**
	↗ progression to regular smoking	**Killen et al. (35)**, **Patton et al. (36)**, **Wang et al. (37)**
Anxiety disorders	↗ smoking rates	Lasser et al. (38), Ziedonis et al. (39)
	↗ nicotine dependence	Piper et al. (40)
	↗ resistance to pharmacotherapy for abstinence	Piper et al. (40)
	↘ rates of abstinence	Piper et al. (41)
	↗ withdrawal symptoms	Weinberger et al. (42)
PTSD symptoms	↗ tobacco dependence	Beckham et al. (43), Thorndike et al. (44), Feldner et al. (45), Greenberg et al. (46)
	↘ rates of quitting and time to relapse after quitting	Lasser et al. (38), Hapke et al. (47), Beckham et al. (48)
	↗ nicotine withdrawal symptoms	Dedert et al. (49)
Schizophrenia	↗ tobacco smoking, nicotine dependence and difficulties to quit	Lasser et al. (38)

*References in bold describe longitudinal studies*.

*ADHD, attention-deficit hyperactivity disorder; PTSD, posttraumatic stress disorder*.

### Impulsivity, Novelty Seeking, and Tobacco Smoking

Impulsivity is a heritable and multifaceted psychiatric construct defined by the tendency to engage in inappropriate, premature, poorly planned, and unduly risky actions without adequate forethought about the potential consequences of this behavior (50–53). It has been associated with drug addiction, including tobacco smoking (54).

Current theories differentiate between motor and cognitive aspects of impulsive behavior. Motor impulsivity reflects a failure in motor inhibition leading to impulsive actions and can be assessed by the ability to exert volitional control over a response that has already been initiated or rendered prominent with extensive training. This type of impulsivity can be notably measured in the “stop-signal reaction time task,” in which subjects are trained to respond as quickly as possible but must inhibit their response when a stop signal is presented, or in a go/no go task (54). While several studies linked deficits in this type of impulsivity with alcohol (55), cocaine (56, 57), and methamphetamine (58) addiction, the data about tobacco addiction are less clear. Thus, tobacco smoking has been shown to decrease inhibitory control in a stop-signal task, where an increased number of errors during the stop signal and increased stop latencies were observed (59). But, another study reported no baseline differences between smokers and non-smokers in the same task (60). In addition, an increase in failure in response inhibition in both stop signal and go/no go tasks was observed after nicotine deprivation in tobacco smokers (61, 62), suggesting that nicotine withdrawal induces deficits in inhibitory control. Interestingly, a recent longitudinal prospective study showed that alterations in neural correlates of response inhibition in adolescents increase the risk for subsequent regular cigarette smoking (15), suggesting that functional brain correlates of response inhibition can be used as a marker of risk for tobacco addiction.

Cognitive aspects of impulsivity include response inhibition, delay discounting, and reward/punishment-based decision-making skills and represent the cognitive processes that regulate impulse control (54, 63–65). The delay discounting describes the tendency to discount the value of a reward as a function of the length of delay to its delivery. Higher delay discounting rates have been associated with cigarette smoking. Thus, current smokers tended to discount future monetary reinforcers more than ex-smokers and non-smokers (66), suggesting that smoking increases cognitive impulsivity in this task and that this effect is reversible. Another study confirmed the increased delay discounting in smokers but found no differences in discounting rates for either money or cigarettes between light and heavy smokers (67), a result confirmed in a recent report (68).

Interestingly, performances in delay discounting at age 10 were shown to predict the initiation of smoking behavior in adolescents at age 14 (12). Also, delay-discounting rate has been identified as a strong prognostic indicator of smoking relapse (13), suggesting that cognitive impulsivity can be a risk factor for subsequent tobacco smoking. Trait impulsivity has also been positively associated with the subjective rewarding effects of nicotine (14) as well as explicit expectancies about nicotine reward (16). A longitudinal study using a sample of college men and women showed that trait impulsivity predicts subsequent smoking initiation (17).

Novelty or sensation seeking can be defined as a heritable tendency to seek out varied, novel, complex, and intense sensations and emotional experiences and to show enhanced behavioral responses to novel situations (69–73). It is one of the most critical individual difference factors predicting drug use among humans (74, 75). Novelty seeking is typically measured in humans by using questionnaires such as the Tridimensional Personality Questionnaire (76), the Zuckerman Sensation Seeking Scale, or the Cloninger’s Temperament and Character Inventory (77). This personality trait was shown to predict tobacco use during adolescence (75, 78) and the early onset of smoking in adolescents (79, 80). In line with this, a study of longitudinal smoking patterns in adolescents found that individuals with high novelty seeking were significantly more likely to become regular smokers than never smokers (18). In addition, novelty seeking was increased in heavy smokers (81) and was positively associated with sensitivity to the initial reinforcing effect of acute nicotine under controlled laboratory conditions (14, 82). A longitudinal study also showed that sensation seeking in college men and women predicts the initiation of smoking and its continuation 20 years later (17). Finally, high levels of novelty seeking have been negatively correlated with smoking-cessation success, with reduced odds of cessation compliance and outcomes (19, 20).

Thus, novelty seeking seems to predict tobacco addiction, but more studies are needed in order to determine the effect of tobacco exposure on this personality trait.

One should nevertheless bear in mind that, although the association between some personality traits and drug addiction is frequently observed, there are no structured and established pre-addictive personalities. Some dissociable personality profiles, including impulsiveness and novelty seeking, may rather be considered as vulnerability factors and facilitate some aspects of the addiction process.

### Attention-Deficit Hyperactivity Disorder and Tobacco Smoking

Attention-deficit hyperactivity disorder is a developmental disorder characterized by hyperactivity, high impulsivity, and an inability to sustain directed attention (83). ADHD affects approximately 6.5–8.4% of children and between 1.9 and 6% of adults (84–86). Evidence suggests that ADHD is a predisposition factor for tobacco smoking. For example, ADHD predicted future smoking (21) and adolescents with ADHD were more likely to experiment with cigarettes and become smokers (22). In addition, ADHD symptoms during childhood, particularly hyperactivity/impulsivity, predicted later nicotine dependence in adulthood (87). ADHD status in childhood was also shown to predict time to relapse to smoking after controlling for gender, history of depression, and baseline smoking variables (23). Smokers with ADHD present an earlier onset of regular smoking, have a higher frequency of smoking behavior, show greater withdrawal symptoms, are more willing to work harder for cigarette puffs, and exhibit a higher level of nicotine dependence than smokers without ADHD (24–29, 88, 89). In addition, there is an increase of ADHD symptoms during periods of abstinence in smokers that was associated with an increased risk of relapse (90). This suggests that the increased withdrawal symptoms observed in ADHD patients negatively affect the success of quitting tobacco smoking. Since ADHD is a neurodevelopmental disorder, there are no data on the influence of tobacco smoking on the emergence of ADHD. However, smoking during pregnancy has previously been strongly associated with the risk of ADHD in offspring (91–95) suggesting a direct causality. However, these studies did not rule out the potential influence of unmeasured familial factors (96, 97), and the association no longer holds in recent studies that used different designs accounting for these factors (97–99). This suggests that maternal smoking during pregnancy reflects a genetic predisposition rather than a causal risk factor for ADHD in offspring. Individuals with ADHD may also be more susceptible to the negative effects of smoking. Thus, smokers exhibited a greater increase in attention deficits over the years than their never-smoking twins (100), suggesting that smoking can worsen attention problems.

In conclusion, there is a complex relationship between ADHD and smoking with ADHD contributing to smoking, but smoking may also contribute to the development of attention deficits.

### Depression and Tobacco Smoking

Depression is characterized by depressed mood, anhedonia, vegetative symptoms, and impaired psychosocial functioning. Cigarette smoking and depression both account for significant morbidity, mortality, and economic burden. Depression is overrepresented among adult smokers and contributes to lower smoking-cessation rates and cigarette smoking is overrepresented in adult smokers prone to depression (101, 102). Longitudinal studies are useful to determine if depressive states can influence tobacco smoking. Thus, a 21-year longitudinal study found an association between major depression (MD) and smoking, with a 19% increase in the average daily smoking rate and a 75% increase in the odds of being nicotine dependent from mid-adolescence to young adulthood (30) in people with MD episode. In addition, adolescents with a history of MD had 50% more risk to progress to daily smoking and were significantly less likely to quit by age 25 compared with controls (31). These results suggest a strong influence of MD on the likelihood to develop tobacco addiction, but several studies suggested that less severe depressive symptoms are also a risk factor for tobacco dependence. For example, depression symptoms at mid-adolescence predicted smoking progression across mid-to-late adolescence (103). Adolescents with higher depressive symptoms were more likely to start smoking (34) and to progress to regular smoking compared with adolescents with lower depressive symptoms (35–37). Another longitudinal study found that depressive symptoms in early adolescence predict faster increases in smoking behavior (104).

In addition, depression seems to have a negative influence on smoking cessation since history of MD reduced the odds of short- and long-term smoking abstinence (32, 33). An increase in negative mood in the early stages of treatment for tobacco dependence was predictive of failure to quit smoking or smoking relapse (105, 106).

These data clearly indicate that depression is a risk factor for tobacco addiction, but other studies also support the opposite, i.e., that smoking influences the development of depression. Thus, cigarette smoking during adolescence was shown to predict the development of depressive symptoms (107–111) and an increased time of smoking dependency has been correlated with increased risk of depression. This suggests that the vulnerability for depression increases with higher rates of smoking (110).

In addition, quitting smoking has been associated with a significant decrease in depression compared with continued smoking (112), supporting the hypothesis that smoking might be the cause for mental health problems and not necessarily the inverse.

In conclusion, despite the fact that some of these studies failed to identify a reciprocal relationship between tobacco addiction and depression (30, 37, 108), the relationship seems to be bidirectional (113). As described earlier, tobacco dependence predicts the development of depressive symptoms and MD, while a history of MD predicts the onset of daily smoking and progression to tobacco dependence. This conclusion is supported by a meta-analysis of 15 longitudinal studies in adolescents that reported evidence for a bidirectional relationship, with a larger effect of depression status on smoking likelihood than the effect of smoking on depression (114).

### Anxiety Disorders and Tobacco Smoking

Anxiety disorders, such as panic disorders, phobias, generalized anxiety disorder, and posttraumatic stress disorder (PTSD), are among the most common mental disorders (115, 116). A strong relationship between anxiety disorders and tobacco smoking has been established in humans. Indeed, while tobacco smoking rates significantly declined from 2004 to 2011 in people without psychiatric illness, this is not the case in people with anxiety disorders (117). Along this line, patients with anxiety disorders had significantly higher smoking rates than a control population (38, 39), and anxiety disorders were significantly more prevalent in people diagnosed with nicotine dependence than in a non-dependent population (118). In addition, patients with social anxiety or generalized anxiety disorders exhibited more severe nicotine dependence at baseline and smokers with a lifetime history of anxiety disorder were resistant to pharmacotherapy for abstinence (40).

PTSD is one of the most common anxiety disorders that can develop in humans after an exposure to one or more traumatic events, with a lifetime prevalence of approximately 8% in the general population (119). Smoking initiation and daily smoking rates were shown to increase after trauma (120, 121), and the presence of PTSD symptoms, such as hyperarousal and emotional numbing, is a predictor of tobacco dependence (43–46). Taken together, these data suggest that anxiety disorders are risk factors for the development of tobacco addiction, but prior smoking has also been found to be associated with increased risk to develop PTSD after a trauma or panic disorder (122, 123). In addition, smoking or smoke exposure in early life increased the likelihood of developing an anxiety disorder later in life (124, 125).

Finally, anxiety disorders have also been associated with greater difficulties for quitting tobacco smoking since smokers with lifetime anxiety disorder have significantly lower rates of abstinence and report more severe withdrawal symptoms than control smokers (41, 42, 126, 127). PTSD patients also exhibited lower rates of quitting, shorter times to first smoking relapse after quitting (38, 47, 48) and experienced worsened nicotine withdrawal symptoms compared with a non-PTSD population (49). However, as for depression, anxiety and stress were shown to be decreased in abstinent subjects by follow-up studies (112). This suggests that the assumption of beneficial effects of nicotine on anxiety and mood, which probably contributes to the maintenance of smoking in populations with mental health problems, should be more drastically challenged to motivate quitting.

Thus, the relationship between anxiety disorders and tobacco addiction is probably bidirectional, a conclusion supported by several additional studies (120, 128–130).

### Schizophrenia and Tobacco Smoking

Schizophrenia is a chronic disabling disorder characterized by positive symptoms (hallucinations and delusions), negative symptoms (blunted affect, alogia, reduced sociability, and anhedonia), and persistent cognitive deficits (memory, concentration, and learning). It affects approximately 1% of the population (131). Cigarette smoking is highly prevalent in persons with schizophrenia and schizoaffective disorder since it ranges from 45 to 88%, compared with <20% in the general population (132). Individuals with schizophrenia smoke more cigarettes per day, are more nicotine dependent, and also have more difficulties in quitting smoking than smokers with no history of mental health problems (38), leading to high mortality due to tobacco-related illnesses (39). Interestingly, smokers with schizophrenia have higher plasma and urine levels of nicotine, even when matched for the number of cigarettes smoked per day and other indices of nicotine dependence (133–135). This is not due to a difference in nicotine metabolism (136) but rather to the manner in which cigarettes are smoked by schizophrenic patients. Indeed, schizophrenic patients take significantly more puffs, have shorter inter puff intervals, and larger total cigarette puff volumes compared with matched healthy control smokers (137). Smokers with schizophrenia also exhibited a higher intensity of demand and greater consumption and expenditure in a cigarette purchase task, suggesting a higher incentive value of cigarettes in smokers with schizophrenia (138).

Thus, schizophrenia appears to be a strong risk factor for tobacco addiction, and individuals with schizophrenia may sustain smoking because of its higher reinforcing effect and to remedy certain symptoms of the disorder (139). Further research is now needed to look at the alternative possibility that tobacco smoking may confer vulnerability to the development of schizophrenia.

## Effects of Nicotine on Cognition, Personality Traits, and Psychiatric Disorders in Humans

As described in the first part of this review, several clinical studies have linked tobacco addiction with impulsivity, novelty seeking, attention, mood disorders, ADHD, and schizophrenia. But, an investigation of the effects of nicotine on these personality traits and psychiatric disorder-associated phenotypes is important to better understand these relationships (see Table 2).

**Table 2:** **Effects of nicotine administration on mental disorder-related processes in clinical studies**.

Mental disorder	Nicotine treatment	Outcome	Reference
Tobacco addiction	Transdermal patch (21 or 35 mg)	↗ attention	Lawrence et al. (140), Hong et al. (141)
	Nasal spray (1 mg)	↗ attention	Warbrick et al. (142)
	Nasal spray (1 mg)	↗ prospective memory	Rusted and Trawley (143)
	Gum (4 mg)	↗ prospective memory	Jansari et al. (144)
Schizophrenia	Transdermal patch (14, 21, or 35 mg)	↗ attention	Barr et al. (145), Hong et al. (141)
	Nasal spray (1 mg)	↗ PPI	Hong et al. (146)
	Subcutaneous injection (12 μg/kg)	↗ PPI	Postma et al. (147)
ADHD	Transdermal patch 7 mg/kg (non-smokers) or 21 mg/kg (smokers)	↘ ADHD symptoms	Conners et al. (148), Levin et al. (149), Bekker et al. (150)
	Transdermal patch (7 mg)	↘ motor impulsivity	Potter and Newhouse (151, 152), Potter et al. (153)
Major depression	Transdermal patch (17.5 mg)	↘ depression symptoms	Salin-Pascual et al. (154)
OCD	Transdermal patch (17.5 mg)	↘ compulsion and anxiety	Salin-Pascual and Basanez-Villa (155)

*PPI, prepulse inhibition of startle reflex; ADHD, attention-deficit hyperactivity disorder; OCD, obsessive–compulsive disorder*.

### Cognition

In addition to its abuse liability, nicotine can also enhance cognitive functions, including attention and memory (156). Thus, nicotine and other nAChR ligands have been proposed as potential therapeutics for the treatment of cognitive deficits in pathologies, such as schizophrenia, ADHD, and Alzheimer’s disease (157, 158). However, chronic cigarette smoking has also been associated with decreased cognitive performance in middle age (159, 160) and increased risk for cognitive decline and dementia later in life (161).

Few studies have investigated the impact of nicotine on attention in humans. For example, transdermal nicotine improved the performance in a rapid visual information-processing task (140, 141) and nicotine exposure trough nasal spray decreased the reaction times in a visual oddball task in smokers (142), suggesting an increase in sustained attention induced by acute nicotine in smokers. Transdermal nicotine also significantly improved attention in both schizophrenic patients and controls (145) and visual attentional performance in mildly deprived smokers (162, 163). These studies clearly indicate that nicotine has a pro-attentional effect in humans. Along this line, there is evidence to suggest that nicotine may be useful in treating the symptoms of ADHD. Thus, positive effects of nicotine have been reported on attention, concentration, and other ADHD symptoms among adults with ADHD (22, 148, 149, 164, 165), indicating that ADHD patients may smoke as a form of self-medication.

Some studies further suggest a promnesic effect of smoking. Thus, abstinent smokers exhibited more impairment in visuospatial working memory (VSWM) compared with current smokers (166), and overnight smoking abstinence in schizophrenic patients’ impaired VSWM performance, an effect reversed by reinstatement of cigarette smoking. The effect of smoking reinstatement was blocked by the non-selective nAChR antagonist mecamylamine (167), indicating that the procognitive effect of tobacco smoking in VSWM tasks is through nAChR activation in patients with schizophrenia. Nicotine administration *via* gum, patch, or injection also improved short-term memory recall in non-smokers (168–170). Interestingly, the effect of nicotine on memory seems to be dependent on baseline performance. Thus, Niemegeers et al. showed that the effect of subchronic nicotine (1 or 2 mg trough oromucosal spray three times daily for 3 days) was dependent on baseline performance in working and visual memory in young and elderly healthy subjects (171). Subjects with lower baseline performance benefited from nicotine administration, while subjects with higher baseline performance performed worse after nicotine administration. This suggests that subjects with lower cognitive performance, irrespective of age, may benefit from nicotine.

There have been few publications on the effect of nicotine on executive functions, and it is difficult to draw conclusions due to the heterogeneity of the procedures and results. For example, nicotine (1 mg through nasal spray) improved prospective memory in minimally deprived (2 h) smokers and non-smokers when the subjects were able to devote resources to that task, but impaired the performance when they completed a concurrent auditory monitoring task (143). Nicotine (2 mg gum) has been shown to improve performance in complex flight simulation tasks, which involve high cognitive load, in non-smoking pilots, but had no effect on the executive function aspect of attention in never smokers (2 and 4 mg gums) (172).

In a study investigating the effect of nicotine on the performance of male non-smokers with high or low attentiveness on the Wisconsin Card Sorting Test (WCST), nicotine administration (7 mg patch) in the high attentiveness group impaired the performance (173). This suggests a deleterious effect of nicotine on strategic planning, set-shifting, and mental flexibility in this sub-population. Finally, in a study using a virtual reality paradigm that assesses multiple cognitive constructs simultaneously (144), nicotine improved the overall performance, time-based prospective memory, and event-based prospective memory in minimally (2 h) deprived smokers (4 mg nicotine gum), but not in never smokers (2 mg nicotine gum). At the same time, action-based prospective memory was enhanced in both groups.

Thus, nicotine seems capable to improve, impair, or have no effect on executive functions depending on the task, the dose of nicotine or the target population, highlighting the need for new studies to obtain a clearer picture on that issue.

Several studies show that cigarette smoking impairs decision-making processes assessed through different neurocognitive tasks (174–177). However, these studies do not discriminate the effects of nicotine alone from the effects other psychoactive compounds found in tobacco smoke. Further studies are needed for providing clear information about the consequences of chronic nicotine exposure on decision-making.

Deficits in pre-attentive sensory information processing, characterized by the inability to filter out or gate sensory information, are thought to contribute to the higher order cognitive deficits observed in schizophrenia. This includes attention, working memory, verbal learning and memory, decision-making, and executive functioning (178, 179). One measure of sensory processing is the P50 suppression that measures the inhibition of electroencephalic cortical response to the second auditory stimulus presented 50 ms after the first. Patients with schizophrenia fail to suppress the response to the second auditory stimulus reflecting gating deficits (180). Several studies have shown that nicotine can improve P50 suppression. Thus, cigarette smoking improved P50 suppression in abstinent smokers with schizophrenia (181), and nicotine gum improved P50 suppression in non-smoking subjects with impaired gating or healthy controls (182–184).

Another measure of sensory information processing is the prepulse inhibition (PPI) of startle reflex that reflects the inhibition of a blinking reflex to a loud startling stimulus presented after a weak prepulse stimulus. This gating mechanism is also impaired in patients with schizophrenia (185) and nicotine (administered *via* nasal spray or subcutaneous injection) improved PPI in smokers and non-smokers with schizophrenia or in healthy subjects (146, 147). In addition, PPI of satiated smokers with schizophrenia is comparable to PPI of smokers without schizophrenia (186). Taken together, these data suggest that nicotine can improve sensory information processing and those patients with schizophrenia may smoke in part to alleviate their deficit in sensory gating.

Very few studies have investigated the effect of nicotine on impulsivity in humans. A positive correlation between levels of nicotine exposure and discounting of delayed monetary reinforcers has been observed in chronic smokers but not in ex-smokers (187, 188), suggesting that nicotine administration trough smoking increases cognitive impulsivity, an effect that is reversible. However, a positive effect of nicotine on the Stop Signal Reaction Time measure of the Stop Signal Task has been observed in adolescent and young non-smoking adults with ADHD, and in a control population (151–153), indicating that nicotine can reduce motor impulsivity. Thus, nicotine appears to have a differential effect on these two types of impulsivity, but more studies are needed to conclude.

We did not find additional clinical data on the effects of nicotine on cognitive impulsivity or on novelty seeking, highlighting the need for such investigations.

### Depression

Self-medication is one of the possible explanations for the impact of depression on cigarette smoking since nicotine reduces negative affect and can have antidepressant effects (189). This theory is supported by the fact that patients with MD increased their smoking behavior when they experienced depressive symptoms (190). In addition, several clinical studies reported that nicotine administration through transdermal patches reduced symptoms of depression, even in non-smoking depressed patients (154, 191) and relieved self-reported depression in regular smokers (150).

Interestingly, chronic administration of low levels of nicotine, as delivered by the nicotine patch, is thought to desensitize, rather than activate, nAChRs (192, 193), suggesting that the therapeutic effect of nicotine on depression may be mediated by inactivation of nAChRs. This is supported by the fact that mecamylamine, a non-selective antagonist at heteromeric nicotinic receptors, decreased depression-like symptoms in patients with Tourette’s disorder (194–197) and enhanced the effects of a selective serotonin reuptake inhibitor (SSRI) in depressed subjects (198).

In conclusion, nicotine can relieve some symptoms of depression, potentially *via* desensitization of nAChRs thus supporting the self-medication hypothesis, which may nevertheless not be the only valid one.

### Anxiety Disorders

Several studies have shown a positive association between symptom severity in PTSD patients and their desire to smoke in order to reduce negative affect (129, 199–201). Other studies also suggested that this association was mediated by the expectancy that smoking would reduce negative affect (202) and that patients with PTSD smoked and relapsed to smoking in response to negative affect and trauma (48, 203). This suggests that people with PTSD smoke to relieve negative affect and anxiety as a form of self-medication, an hypothesis supported by the fact that PTSD symptoms are reduced by nicotine intake (43–46) and by the anxiolytic effect of nicotine patches in non-smokers with obsessive–compulsive disorders (155). Thus, people with anxiety disorders may smoke to alleviate their symptoms, but more clinical studies on the effect of nicotine on anxiety are needed to support this conclusion.

## Predisposing Endophenotypes for Nicotine Taking and Seeking in Preclinical Studies

Some psychological constructs, in particular, have been repeatedly associated with vulnerability to addiction, e.g., sensation seeking, impulsivity, and anxiety (6, 7, 204, 205). To date, the majority of preclinical animal research on individual differences in the response to drugs of abuse has mostly focused on cocaine. Additional work is now needed for nicotine, although some interesting data have nevertheless been generated as detailed in the following paragraphs (see Table 3). In this review, we will strictly focus on behaviors reflecting processes that directly contribute to the addiction cycle, such as those related to (i) drug rewarding properties (e.g., conditioned place preference (CPP), acquisition of self-administration), (ii) later stages of self-administration (e.g., increasing fixed ratios), (iii) motivation for the drug (e.g., progressive ratio schedules of reinforcement), (iv) persistence of drug seeking (e.g., extinction of self-administration), (v) relapse, and (vi) withdrawal syndrome during abstinence.

**Table 3:** **Association between pre-existing endophenotypes and nicotine addiction-related features in animal studies**.

Pre-existing phenotype	Nicotine addiction-related features	Species	Reference
Motor impulsivity (5-CSRTT)	↗ IVSA acquisition and under PR schedule	Wistar rats	Diergaarde et al. (206)
Cognitive impulsivity (delayed discounting task)	↗ IVSA under PR schedule	Wistar rats	Diergaarde et al. (206), Diergaarde et al. (207)
	↗ resistance to extinction of nicotine-seeking after IVSA		
	↗ cue-induced reinstatement of nicotine seeking		
	Ø somatic withdrawal	Lister-hooded rats	Kolotroni et al. (208)
Locomotor response to novelty (horizontal locomotion)	↗ IVSA acquisition and under PR schedule	Sprague-Dawley rats	Suto et al. (209)
	Ø IVSA acquisition and under PR schedule	Sprague-Dawley rats	Guillem et al. (210)
	↘ nicotine-induced CPP	C57Bl/6N mice	Bernardi and Spanagel (211)
	↘ nicotine-induced CPP	Sprague-Dawley rats	Pastor et al. (212)
	↗ social anxiety in response to a nicotine challenge after nicotine abstinence	Sprague-Dawley rats	Aydin et al. (213–216)
Locomotor response to novelty (rearing) and novelty seeking (novel object preference)	Ø voluntary oral nicotine intake and nicotine-induced CPP	Wistar rats	Pawlak and Schwarting (217, 218)
Novelty seeking (novel object preference)	Predictive of nicotine IVSA	Sprague-Dawley rats	Wang et al. (219)
Novelty seeking (hole-board activity box)	↗ voluntary oral nicotine intake	C57Bl/6 mice	Abreu-Villaca et al. (220)
Anxiety (EPM and hole-board activity box)	Ø voluntary oral nicotine intake	C57Bl/6 mice	Abreu-Villaca et al. (220), Manhaes et al. (221)
Anxiety (EPM)	Ø voluntary oral nicotine intake	Wistar rats	Pawlak and Schwarting (217)
Anxiety (CPP apparatus used as a dark–light box)	↗ nicotine-induced CPP	Sprague-Dawley rats	Falco et al. (222)
Anxiety (EPM)	Predictive of nicotine IVSA and context-induced reinstatement of nicotine seeking	Sprague-Dawley rats	Wang et al. (219)
Stress reactivity (multiple tests)	Ø IV SA acquisition and extinction of nicotine seeking	Intercross between C57Bl/6J and C3H mice	Bilkei-Gorzo et al. (223)
	↗ stress-induced reinstatement of nicotine seeking		
Depression (tail suspension test)	Predictive of nicotine IVSA and context-induced reinstatement of nicotine seeking	Sprague-Dawley rats	Wang et al. (219)

*5-CSRTT, 5-choice serial reaction time task; IVSA, intravenous self-administration; PR, progressive ratio; CPP, conditioned place preference; EPM, elevated plus maze*.

### Impulsivity

High impulsivity has been associated with a wide range of neuropsychiatric disorders, including ADHD (224), mood disorders (225), and also drug addiction (64, 226, 227). Findings in trait-impulsive laboratory animals suggest that high impulsivity represents a vulnerability factor for addiction to several classes of drugs including cocaine (228–230), alcohol (231), and nicotine (53, 206). One plausible hypothesis is that high impulsivity results from a dysfunction of the frontal cortex and that this pre-existing dysfunction may facilitate the progressive incapacity of the frontal cortex to suppress maladaptive responses that develop following repeated exposure to a drug (232). Alternatively, drug intake may normalize excessive impulsivity in some individuals and may therefore represent a form of self-medication (53). As described earlier, impulsivity encompasses a complex array of behavioral processes, which can be categorized through at least two major components: motor/action impulsivity (motor disinhibition) and cognitive/choice impulsivity (impulsive decision-making). Several procedures have been developed to provide objective measures of impulsivity in animals, including delay-discounting tasks and the 5-choice serial reaction time task, an analog of the human continuous performance task (233, 234).

Very few preclinical studies have examined the putative link between pre-existing manifestations of impulsivity and nicotine addiction-like behaviors. Yet, one comprehensive study has shown that poor impulse control influences the motivational properties of nicotine and of nicotine-associated cues on a self-administration procedure in rats, and that sub-dimensions of impulsivity predict vulnerability to distinct stages of nicotine-seeking behavior (206). The authors found that high motor impulsivity on a 5-choice serial reaction time task predicts both enhanced self-administration of nicotine during the acquisition and increased motivation for nicotine under progressive ratio of reinforcement. At the same time, high choice impulsivity on a delayed reward task was mostly predictive of both increased resistance to extinction of nicotine-seeking and increased cue-induced relapse of nicotine seeking after extinction. High-impulsive choice was also associated with higher motivation for nicotine when ratios of response requirement are increased, an observation that was confirmed by these authors in the second study (207). In contrast, high- and low-impulsive rats selected on a delay discounting task appear to show similar somatic withdrawal syndrome intensity after chronic exposure to low dose of nicotine (208). These data suggest that the two sub-dimensions of impulsivity influence both distinct and overlapping processes through the dynamics of addiction development in vulnerable individuals.

### Response to Novelty

The second behavioral factor strongly linked to addiction including smoking is the novelty/sensation seeking trait (7, 205, 235). Like impulsivity, novelty/sensation seeking represents a multifaceted behavioral construct and can be divided into a number of dimensions. Several tasks have been developed in animal models to assess responses to novelty.

The primary animal model of sensation seeking is measured as an enhanced locomotor activity in a novel and inescapable environment (236, 237). As for impulsivity, only a small number of preclinical studies have examined the relationship between pre-existing high locomotor response to novelty and nicotine addiction-like behaviors. Consistent with what was reported for other psychostimulants (237), one study found that high locomotor responding to a novel environment predicted the propensity to self-administer nicotine under both fixed and progressive ratios of reinforcement in rats (209). However, such an association was not observed in a more recent study where rats screened as high and low responders to novelty displayed similar levels of nicotine self-administration, although high responders were more prone to self-administer nicotine when it was delivered concomitantly with IMAOs (210). In contrast, a study reported that mice showing low basal locomotor activity manifested nicotine-induced CPP, while mice exhibiting high basal locomotor activity did not (211). However, in this study, the mice had previously been exposed to nicotine for prior experimental testing, which might have influenced subsequent nicotine rewarding effects (238). Consistently, other authors showed that rats classified as low responders according to their locomotor response to novelty following an injection of nicotine, showed nicotine-induced CPP after a long- but not short-term conditioning procedure, while rats classified as high responders did not show CPP under any condition (212). Also, rats selected as high locomotor responders to novelty showed enhanced social anxiety-like behavior during abstinence after repeated nicotine exposure (213–216).

In addition to the sensation seeking trait that is modeled as high locomotor reactivity to novel environments, novelty seeking has been proposed to reflect a distinct dimension of sensation seeking that would differentially contribute to the vulnerability to develop addiction (239, 240). The terms sensation seeking and novelty seeking are often used in an exchangeable way throughout the literature, though. In animal studies, novelty seeking *per se* is modeled by a high propensity to visit a novel object or environment in a free choice procedure, the so-called novelty preference. Very few studies have attempted to identify the predictive value of novelty seeking to the appetence for nicotine. Interestingly, it has been shown that rats, screened as high novelty seekers as measured by their preference for a novel object in a procedure where they could freely explore either a novel or a familiar object, were also characterized as high locomotor responders to novelty as measured by the number of rears they displayed in an open-field (217). However, high novelty seeker rats did not show differences compared with rats screened as low novelty seekers when subsequently tested for oral nicotine consumption. In another study, the same authors also observed no enhanced nicotine-induced CPP in rats with high rearing activity, although it is difficult to conclude since they did not observe nicotine CPP in any of the rat subpopulations tested in this study (218). Using multiple regression analysis, other authors reported that novelty seeking measured as exploration of a novel object predicted nicotine self-administration in female, but not in male, rats (219). Another animal model of novelty seeking based on the number of head-dips in the hole-board apparatus has been used (241). Mice preselected for high novelty seeking in this test showed a marked increase for oral nicotine intake over time, while mice with low novelty seeking did not (220). However, mice showing high head-dip behavior in the hole-board task and that had been exposed to nicotine during gestation and suckling tended to consume less nicotine when tested during adolescence (242). In contrast, the same study showed that mice similarly exposed to nicotine and showing high rearing or high general locomotor behavior in the hole-board displayed increased oral nicotine intake.

Taken together, these data suggest that additional work is clearly needed to conclusively acknowledge whether high response to novelty/high novelty seeking represents a significant risk factor for nicotine addiction and, if so, for which specific features of this disorder. Novelty seeking measured as high novelty preference, but not high novelty-induced locomotor activity, has notably been shown to predict the compulsive use of cocaine in rats, a hallmark feature of addiction (243). The existence of a similar causal association has not been investigated for nicotine, partly because behaviors reflecting loss of control over nicotine intake and compulsive nicotine taking and seeking have not been accurately modeled so far. The recent development of increasingly reliable models may open new paths for such longitudinal investigations (244–247).

### Anxiety and Mood Disorders

There is a high prevalence of tobacco smoking in subjects with mood or anxiety disorders (235, 248–250). It has been proposed that individuals may use drugs including nicotine as a coping strategy to self-regulate affective distress states (251–253). Drug users may self-medicate for affective distress existing before the initiation of drug use and also to alleviate mood and anxiety distress that are part of the withdrawal syndrome resulting from abstinence (254). Alternative explanations for the strong association between smoking and mood and anxiety disorders are also to be considered, notably since repeated use of nicotine significantly impacts anxiety and mood processing. Below, we review the preclinical studies that assessed whether the manifestation of such disorders beforehand may predict the future response to nicotine.

In preclinical studies, anxiety is usually assessed using procedures that exploit the emotional conflict occurring between the innate strong tendency to explore novel environments and the natural fear of open and/or brightly lit spaces. In particular, the elevated plus maze (EPM) is commonly used with anxiety measured as the preference of animals for closed versus open arms (255). High anxiety in this task predicts several features of cocaine and alcohol, but not heroin, addiction (7). Adolescent mice with high anxiety in this test showed similar levels of oral nicotine intake as mice with low anxiety in a free choice procedure (221). However, during a withdrawal period after 2 weeks of exposure to nicotine through their drinking bottles, adolescent mice with high anxiety consumed less nicotine than mice with low anxiety when tested in a free choice procedure (221). The same group further showed no differences in oral consumption of nicotine in a free choice procedure between adolescent mice with high and low anxiety classified according to their percentage of center squares crossed in a hole-board activity box (220). Another study also reported no association between prior behavioral measurements on the EPM and oral nicotine consumption in rats (217). In contrast, a study in adolescent rats reported that individuals with high anxiety measured as the time spent in the white versus the black chamber of a biased CPP apparatus manifested subsequent nicotine-induced CPP while individuals with low anxiety did not (222). Furthermore, in a comprehensive study assessing several risk factors for nicotine self-administration in a social context in rats, multiple regression analysis found that anxiety measures on the EPM were a predictor of nicotine intake in males, but not in females, while measures of depression on the tail suspension test were predictors of nicotine intake in both males and females (219). In males, both depression- and anxiety-related measures also predicted context-induced nicotine reinstatement. Interestingly, mice generated from the intercross of high (C57BL/6J) and low (C3H/J) emotional mouse strains and classified as “high stress reactive” according to their scores in an elevated zero maze, light–dark box, startle response, and forced swim tests, showed higher vulnerability to relapse but not to initiation or maintenance of nicotine self-administration compared with low and average stress reactive animals (223).

In addition to data regarding the causal link between inter-individual differences in anxiety- and depression-like behaviors and appetence for nicotine, it was demonstrated that acute stressor exposure through a single episode of intermittent footshock administered 24 h before the start of place conditioning dose-dependently facilitated acquisition of CPP to nicotine in adolescent rats (256). Prenatal stress in rats also increased nicotine reinforcing properties in a CPP procedure and anxiety withdrawal symptom at the cessation of nicotine exposure (257, 258). Finally, chronic mild stress, considered as a model of depression, which was delivered prior to nicotine exposure was found to exacerbate nicotine withdrawal syndrome in rats (259).

Although these data are heterogeneous, they suggest that anxiety and mood disorders may represent a significant predictor of nicotine addiction and may notably influence the vulnerability to relapse after abstinence, depending on the sex and the age of the individual.

### Cognitive Impairments

In addition to alleviating stress, anxiety, and improving mood, nicotine has the ability to enhance cognition. Nicotine use has also been proposed as a self-treatment for cognitive deficits that are encountered in numerous psychiatric diseases strongly represented in smoker populations such as schizophrenia or ADHD (260). As for other aspects of the comorbidity between smoking and psychiatric conditions, one fundamental pending question is whether cognitive deficits are of premorbid origin or develop after long-term exposure to nicotine and subsequent withdrawal. Animal models have proven to be useful tools for helping to resolve these issues with the possibility for well-controlled longitudinal studies to be conducted. Nevertheless, while many studies have looked at the effects of nicotine on cognitive processes, there is a great lack of preclinical studies investigating the relationship between inter-individual differences in cognitive functions, such as baseline impairments in attention, learning, and memory functions, and addiction-like behaviors, especially with regard to nicotine. One study provided evidence for a causal link between prior cognitive deficits and behavioral response to nicotine, by looking at individual differences in baseline PPI of acoustic startle reflex and subsequent nicotine-induced locomotor effects including locomotor sensitization. Disruption in the PPI is a model of cognitive impairment in schizophrenia and reveals deficits in the sensorimotor gating system which is critical for the integration of sensory and cognitive information processing and execution of appropriate motor responses. The authors showed that the acute effect of nicotine on locomotion was higher in rats classified as high-inhibitory, while a locomotor sensitization after repeated exposure to nicotine developed only in low-inhibitory rats (261). Another study reported that neonatal ventral hippocampal lesions that produced post-adolescent onset, pharmacological, neurobiological, and cognitive features of schizophrenia, such as spatial learning and working memory deficits, increased nicotine self-administration and nicotine seeking during extinction in adult rats (262). Furthermore, spontaneously hypertensive rats, considered as the most valid animal model of ADHD and that display symptoms of inattentiveness, impulsivity, and hyperactivity, show enhanced nicotine self-administration (263) and CPP (264). It has also been shown that social interaction phenotypes are predictor of nicotine self-administration and nicotine seeking in rats, although it is difficult to conclude about which cognitive functions – if any – were implicated in such a causal association (219).

Taken together, these data suggest that different behavioral factors may preferentially contribute to some of the many dimensions of the addiction cycle. Combinations of some predisposing behavioral traits may result in specific vulnerability profiles predicting higher risk for starting nicotine use or shifting toward nicotine abuse, or for relapse during abstinence. For instance, outbred rats classified as high locomotor responders to novelty show decreased anxiety as compared with low responders (265). Also, as mentioned earlier, a study based on a dimensional analysis approach within a single and large population of rats reported that high locomotor reactivity to novelty predicts the propensity to self-administer cocaine, while high novelty seeking in a free choice procedure predicts the transition to compulsive cocaine seeking (243). Additional studies measuring the inter-individual vulnerability for different personality traits and addiction-like phenotypes in the same population of animals may significantly improve our understanding of vulnerability to nicotine addiction.

## Effects of Nicotine on Cognitive and Affective Endophenotypes in Preclinical Studies

### Impulsivity

In addition to a possible influence of pre-existing impulsivity on later development of drug abuse, psychostimulant abuse may itself lead to the increased impulsivity often observed in chronic drug abusers, including nicotine, and, thereby, help to develop and maintain addiction (see Table 4) (348).

**Table 4:** **Effects of nicotine administration on affective and cognitive processes in animal studies**.

Phenotype	Nicotine treatment	Outcome	Species	Reference
Motor impulsivity (serial reaction time-; go/no go-; stop-signal-; and DRL-tasks)	Acute	↗	Lister-hooded rats Sprague-Dawley rats Wistar rats	Mirza and Stolerman (266) Stolerman et al. (267) Blondel et al. (268), Bizarro et al. (269), van Gaalen et al. (270), Semenova et al. (271), Tsutsui-Kimura et al. (272), Kolokotroni et al. (273)
	Chronic	↗	Sprague-Dawley rats Lister-hooded rats Wistar rats	Blondel et al. (268) Grottick and Higgins (274) Semenova et al. (271), Counotte et al. (275), Kirshenbaum et al. (276)
		Ø	Wistar rats	Counotte et al. (275)
Cognitive impulsivity (delayed discounting task)	Acute	↗	Wistar rats Lister-hooded rats Long–Evans rats	Dallery and Locey (277) Kolokotroni et al. (273) Kelsey and Niraula (278)
		↘	Fischer rats Lewis rats	Anderson and Diller (279)
	Chronic	↗	Long–Evans rats	Dallery and Locey (277), Kelsey and Niraula (278)
		Ø	Wistar rats Fischer rats Lewis rats	Counotte et al. (275) Anderson and Diller (279)
Anxiety-like behaviors (EPM; social interaction test; open field; dark–light box)	Acute	↘	Sprague-Dawley rats Lister-hooded rats CD1 mice C57Bl/6 mice BALB/C mice	O’Neill and Brioni (280) File et al. (281) Irvine et al. (282) Balerio et al. (283, 284) Villegier et al. (285), McGranahan et al. (286), Varani et al. (287)
		↗	Lister-hooded rats Wistar rats CD1 mice BALB/C mice Swiss mice	File et al. (281) Ouagazzal et al. (288) Irvine et al. (282) Irvine et al. (289) Balerio et al. (283, 284), Biala and Kruk (290), Biala et al. (291), Zarrindast et al. (292), Varani et al. (287)
		Ø	Lister-hooded rats Sprague-Dawley rats	Ouagazzal et al. (288) Villegier et al. (285)
	Chronic	↘	Wistar rats Lister-hooded rats Sprague-Dawley rats Swiss mice	Ericson et al. (293) Irvine et al. (289) Elliott et al. (294) Biala and Kruk (290), Biala et al. (291)
		↗	Wistar rats Sprague-Dawley rats C57Bl/6J mice	Irvine et al. (295) Elliott et al. (294) Caldarone et al. (296), Trigo et al. (297), Bura et al. (298)
		Ø	Wistar rats C57Bl/6J mice	Besson et al. (299) Ijomone et al. (300), Caldarone et al. (296)
Fear conditioning/contextual safety discrimination	Acute	↗	C57Bl/6 mice BALB/C mice A/J mice 129/SvEv mice DBA/1J mice DBA/2J mice	Gould and Wehner (301) Gould (302) Gould and Higgins (303) Gould and Lommock (304) Wehner et al. (305) Davis et al. (306), Davis et al. (307), Portugal et al. (308)
		Ø	C57Bl/6 mice C3H/HeJ mice CBA/J mice	Gould and Wehner (301) Gould (302) Portugal et al. (308)
		↘	Wistar rats C57BL/6J	Szyndler et al. (309) Kutlu et al. (310)
	Chronic	Ø	Wistar rats C57Bl/6 mice BALB/C mice A/J mice 129/SvEv mice DBA/1J mice DBA/2J mice C3H/HeJ mice CBA/J mice	Szyndler et al. (309) C57Bl/6 mice BALB/C mice A/J mice 129/SvEv miceDavis et al. (306) Portugal et al. (308)
Depression-like behaviors (learned helplessness; forced swim-; and tail suspension tasks)	Acute	↘	Sprague-Dawley rats Wistar rats Flinders sensitive line rats Fawn-hooded rats C57Bl/6J mice BALB/C mice	Tizabi et al. (311) Vazquez-Palacios et al. (312) Suemaru et al. (313) Andreasen and Redrobe (314) Tizabi et al. (315) Villegier et al. (285)
		Ø	Sprague-Dawley rats Flinders resistant line rats ACI/N rats NMRI mice	Tizabi et al. (311) Andreasen and Redrobe (314) Tizabi et al. (315) Villegier et al. (285)
	Chronic	↘	Wistar rats Flinders sensitive line rats Flinders resistant line rats Fawn-hooded rats Wistar-Kyoto rats	Semba et al. (316) Djuric et al. (317) Tizabi et al. (311) Vazquez-Palacios et al. (312) Tizabi et al. (315), Tizabi et al. (318)
		Ø	Wistar rats Flinders resistant line rats ACI/N rats	Tizabi et al. (311) Tizabi et al. (315) Tizabi et al. (318), Ijomone et al. (300)
		↗	Wistar-Kyoto rats	Tizabi et al. (318)
Learning and Memory (active/passive avoidance learning; radial-arm maze; Lashley III maze; object recognition task; water maze; serial pattern learning)	Acute	↗	Wistar rats Sprague-Dawley rats	Puma et al. (319) Levin et al. (320), Levin et al. (321)
		Ø	Sprague-Dawley rats NMRI mice	Levin et al. (320) Moragrega et al. (322)
		↘	NMRI mice	Moragrega et al. (322)
	Chronic	↗	Sprague-Dawley rats Fischer rats CD1 mice	Levin et al. (323) Levin et al. (324) Levin et al. (325), Arendash et al. (326), Socci et al. (327), Levin and Torry (328), Yilmaz et al. (329), Attaway et al. (330), Levin et al. (331), Ciamei et al. (332)
		↘	Sprague-Dawley rats	Yilmaz et al. (329)
		Ø	Sprague-Dawley rats Fischer rats NMRI mice	Levin and Torry (328) Attaway et al. (330) Vicens et al. (333)
Attention (5-CSRTT; 2-choice stimulus detection task)	Acute	↗	Lister-hooded rats Wistar rats Sprague-Dawley rats Fischer × Brown Norway hybrid rats	Mirza and Stolerman (266) Blondel et al. (334) Grilly (335) Grilly et al. (336), Mirza and Bright (337), Bizarro and Stolerman (338), Quarta et al. (339), Semenova et al. (271)
		Ø	Wistar rats Lister-hooded rats	Mirza and Bright (337) Semenova et al. (271)
	Chronic	Ø	Wistar rats	Blondel et al. (334)
		↗	Lister-hooded rats Wistar rats	Grottick and Higgins (274) Hahn and Stolerman (340), Hahn et al. (341), Semenova et al. (271)
Probability discounting	Acute	↘ or Ø	Long–Evans rats	Mendez et al. (342)
	Chronic neonatal	Ø	Sprague-Dawley rats	Mitchell et al. (343)
Reversal learning	Chronic	↘	C57Bl/6J mice	Ortega et al. (344), Cole et al. (345)
		Ø	C57Bl/6J mice	Ortega et al. (344)
Strategy shifting	Chronic	Ø	C57Bl/6J mice	Ortega et al. (344), Cole et al. (345)
Attentional set-shifting	Acute	↗	Lister-hooded rats	Allison and Shoaib (346), Wood et al. (347)
	Chronic	↗	Lister-hooded rats	Allison and Shoaib (346)

*DRL, differential reinforcement of low rate; EPM, elevated plus maze; 5-CSRTT, 5-choice serial reaction time task*.

Animal studies on the effects of nicotine on inhibitory control have mostly focused on motor impulsivity using attentional tasks. Acute nicotine exposure consistently increased premature responding on serial reaction time- (266–272) and go/no-go-tasks in rats (273). These effects appear to be long-lasting, although data about chronic exposure to nicotine on motor impulsivity are fewer and less consistent (268, 271, 274, 276). One recent study in mice demonstrated that chronic oral, but not acute, injections of nicotine attenuated phencyclidine-induced increases in motor impulsivity (349). Increased motor impulsivity was further reported in rats after prenatal exposure to nicotine, while cognitive impulsivity was not affected (350, 351). In adolescent, but not post-adolescent rats, repeated exposure to nicotine increased impulsive action but not impulsive choice (275).

Few animal studies have focused on the consequences of nicotine exposure on cognitive impulsivity using delay-discounting tasks, and the data are more heterogeneous. Acute injections of nicotine dose-dependently increased impulsive choice in rats, while repeated injections of nicotine also increased impulsive choice, but to the same extent regardless of the dose (277). After nicotine treatment cessation, impulsive choice remained enhanced for a long period before gradually returning to baseline, suggesting that chronic nicotine exposure can produce long-lasting although reversible alterations in inhibitory control. Acute exposure to nicotine increased both impulsive action in a go/no go task and impulsive choice in a delayed reward task in rats, with greater sensitivity of impulsive choice to nicotine (273). Both acute and subchronic injections of nicotine increased impulsive choice in rats in a procedure where the delayed reward was made preferable by decreasing the probability rather than the magnitude of the immediate reward (278). In contrast, a study reported decreased impulsive choice in rats after acute nicotine, and this effect was abolished after repeated nicotine injections (279). Finally, in rats with high cognitive impulsivity, chronic nicotine exposure and nicotine withdrawal had no effect on impulsive choice, while chronic nicotine exposure increased impulsive choice in low-impulsive rats, with no effects on animals with intermediate impulsivity levels (352). Nicotine may result in varying effects on choice processing, depending on key parameters such as basal levels of impulsivity, reinforcement amount, or delay (e.g., adjusting versus fixed delay), and genetic background of rats.

### Anxiety and Mood Disorders

The effects of acute nicotine exposure on anxiety-like behavior is highly dependent on the task, dose, timing of testing, sex, strain, age, and basal anxiety levels of the animals (353, 354). In the EPM, acute or subchronic systemic nicotine was found anxiolytic in some studies (280, 285, 293), anxiogenic at both low and high doses in others (288, 289, 292, 294), or to have no effects (288), in rats. Inconclusive data have also been obtained in mice, with anxiolytic effects at low doses and anxiogenic effects at high doses of nicotine in C57BL/6J, CD1, and BALB/C mice (283, 284, 286, 287), and anxiogenic effects with an intermediate dose with anxiolytic action when given subchronically in Swiss mice (290, 291). In the social interaction test, it is also generally found that low doses of nicotine induce anxiolytic effects, while high doses are anxiogenic (281). However, a study reported that acute nicotine injections performed 5 min before testing induced anxiogenic effects, whereas nicotine injections using the same dose but performed 30 min before the task elicited anxiolytic effects (282). Nicotine reduced stress-induced hyperthermia (355).

Interestingly, a tolerance to nicotine’s effects on anxiety may develop over time. Chronic exposure to nicotine was found to have no longer effects on anxiety or to induce anxiolytic effects to which tolerance also develops eventually in the EPM and the social interaction test, in rats and mice (289–291, 293, 299, 300). The consequences of chronic nicotine exposure also depend on several factors such as sex or basal levels of anxiety. For instance, mice that overexpress the R isoform of acetylcholinesterase exhibit increased anxiety that is normalized by chronic forced nicotine consumption (356). Chronic nicotine treatment also reversed affective deficits produced by chronic mild stress (357). Yet, increased anxiety was also observed in the EPM and the light–dark box after chronic nicotine consumption (296–298). One study reported increased anxiety in the social interaction test in rats after nicotine self-administration, which may appear contradictory to the self-medication hypothesis (295).

Increased anxiety is consistently observed when testing is performed during nicotine withdrawal in the EPM, light–dark box, or social interaction test (221, 282, 295, 358–361) and is reduced by nicotine injection (289). Nicotine withdrawal also increased sensitivity to stressors in the light-enhanced startle paradigm (362).

These studies suggest that nicotine effects on anxiety are dependent on various factors such as the source of anxiety, baseline levels, and genetic background of the individuals. Nicotine may be used to self-medicate anxiety-related distress associated with abstinence or in people with predisposing phenotypes, while it may have opposite effects on anxiety in other individuals or under different conditions. In the latter case, smoking behavior might be sustained by the belief that nicotine consumption will alleviate the anxiety that was essentially induced by smoking itself in the first place, while long-term smoking cessation would actually be much more beneficial for reversing such anxiety-related problems.

The effects of nicotine on fear conditioning in rodents are clearer than those on anxiety-like behavior (363). Studies have consistently reported enhanced hippocampus-dependent fear conditioning in mice after acute nicotine exposure (302–305, 307), while there is no effect on hippocampus-independent fear conditioning or on general freezing behavior (301, 302). Acute nicotine was further shown to impair contextual safety discrimination in a safety learning paradigm (310). A tolerance to these effects seems to develop under chronic nicotine exposure in mice and rats, while nicotine withdrawal altered fear conditioning (306, 308, 309, 353, 363). Furthermore, a study showed that nicotine had differential effects on extinction of fear conditioning depending on when it was administered, during training and/or during extinction, and on the context during extinction (364), suggesting that nicotine may strengthen contextual fear memories and interfere with extinction. Chronic nicotine administration 2 weeks prior to the training impaired subsequent cued – but enhanced contextual – fear extinction (365). Studies on fear conditioning extinction are particularly relevant in the context of the self-medication for emotional distress hypothesis of nicotine abuse. Further investigation will hopefully be carried along this line in the future.

Numerous studies showed antidepressant-like effects of nicotine in rat and mouse models, such as in learned helplessness (316) and forced swim tests (311–314, 317). However, some authors have observed decreased depression-like phenotypes in response to nicotine only in rat strains that display enhanced basal levels of depressive features, with contradictory effects depending on the post-injection time of the testing (311, 315, 318). As for anxiety, factors including age, sex, and genetic background may also influence the action of nicotine on mood. One study notably demonstrated that while acute nicotine decreased depression-like behavior in adult Sprague-Dawley rats, it had no effect in adolescent rats (285). There is also evidence for decreased depression-like phenotypes following chronic nicotine exposure (312, 316). Furthermore, chronic administration of nicotine results in an enhanced response to classical antidepressants (314, 366) and reverses anhedonia induced by chronic stress (367). Acute and chronic exposure to nicotine also had antidepressant effects in environmentally induced rat models of depression (357, 368, 369). Interestingly, chronic oral nicotine intake or repeated nicotine injections diminished depressive symptoms more than transcranial magnetic stimulation (369). However, one study found no depression-like phenotypes in response to chronic nicotine in the tail suspension task in male and female rats, whatever the dose of nicotine tested (300). By contrast, nicotine withdrawal is clearly associated with enhanced depression-like behaviors, including elevated reward thresholds (370) in rats. At early stages of withdrawal, mice exhibited a depression-like profile similar to that observed following a chronic stress regimen (367). Acute administration of the antidepressant fluoxetine reversed nicotine withdrawal-induced intra-cranial self-stimulation threshold elevations when coadministered with a 5-HT1A receptor antagonist (371).

Overall, there is evidence supporting the self-medication hypothesis for anxiety and depressive-like symptoms, including those resulting from nicotine exposure cessation. Subsequent nicotine-seeking relapse may be driven by negative reinforcement mechanisms that anticipate such affective distress (260). However, nicotine-elicited improvements of anxiety and mood appear to strongly depend on several conditions. Nicotine can also deteriorate affective states in some conditions, an important fact that may paradoxically contribute to smoking maintenance and should be taken in account to provide appropriate smoking cessation help.

### Cognitive Impairments

Accumulating evidence suggests that cognitive enhancement may contribute to nicotine addiction through different modalities. Research using experimental animals has provided a better understanding of the effects of nicotine on cognitive processes.

Nicotine administration has been shown to improve learning and memory (157, 319, 321, 329, 331, 372, 373). Single injections of nicotine notably improved working memory in rodents (157, 320). Acute nicotine administration also enhanced acquisition, consolidation, and restitution of the information in an object recognition task in rats (319). Yet, it was reported that acute nicotine did not improve acquisition in the water maze in group housed mice and even impaired performances in this task in individually housed mice (322). Importantly, many preclinical studies show that the efficacy of nicotine on memory does not diminish with chronic administration. For instances, chronic nicotine exposure improves memory performances in rats (323–326) or memory consolidation in mice (332). Nevertheless, some studies found no effects of chronic administration on memory function. Notably, chronic nicotine in NMRI male mice did not significantly change performance in the water maze (333). Age may be a significant factor influencing the action of nicotine on memory. A study reported that nicotine improved the acquisition of a serial pattern learning task in young but not old Fisher 344 rats, while no effects were found on reference memory in either group (330). Chronic nicotine administration also failed to improve working memory in old rats (328). Yet, other studies obtained contrasting data with improvements of memory in response to nicotine in senescence-accelerated mice (374) and aged rats (327). Nicotine also alleviated memory deficits induced by chemical or pharmacological agents (375, 376), and brain lesions (377, 378). By contrast, nicotine withdrawal resulted in learning and memory impairments including in contextual fear conditioning (306, 379, 380).

Although these data suggest primary mnemonic effects of nicotine, there has been much debate as to whether beneficial effects of nicotine in tasks of learning and memory may be secondary to effects on attentional functions. A first study reported that small doses of nicotine reversed deficits in 5-CSRTT accuracy in basal forebrain lesioned rats, but not in non-lesioned animals (381). Nevertheless, other studies showed improvements in 5-CSRTT response accuracy following acute (266, 334, 338, 339) and chronic (271, 274, 340, 341) exposure to nicotine, although these effects may be strain dependent (337). Nicotine also induced improvements in choice accuracy in a two-choice stimulus detection task (335, 336). As observed for learning and memory, nicotine reversed attentional impairments caused by brain or pharmacologically induced lesions (325, 381, 382). Nicotine withdrawal was shown to impair choice accuracy, to increase omission errors in the 5-CSRTT (271, 383), and to impair PPI of acoustic startle in mice (384), although contrasting results were found with another stain of mice (385).

Apart from learning, memory, and attention functions, very few studies have focused on the consequences of nicotine exposure on executive functions in animals. Some studies have evaluated the effects of nicotine on measures of cognitive flexibility. Deficits in cognitive flexibility may contribute to drug addiction as the inability to change a response to stimuli previously associated with a drug stimulus or reward (386). Acute nicotine injections impaired decision-making, and this effect was associated with deficits in behavioral flexibility measured as perseverating responding in rats (342). The same authors reported that chronic neonatal nicotine did not impair decision-making in rats (343). Yet, chronic exposure to a high, but not low, dose of nicotine impaired response reversal learning in mice (344, 345). In contrast, other authors (346, 347) reported that acute and repeated nicotine administration improved attentional set-shifting in rats.

## Conclusion

The studies related across this review strongly support the idea that inter-individual differences in cognitive and affective processing both preceding and resulting from repeated exposure to nicotine contribute to nicotine addiction. There is growing evidence that nicotine addiction arises from the combined interactions of various processes underlying cognition and emotion with nicotine exposure according to several modalities.

First, human studies, but mostly preclinical investigations, clearly indicate that nicotine can have direct facilitator effects on cognitive processing and alleviate negative affective states, supporting the hypothesis of tobacco smoking as a form of self-medication. This seems to be particularly the case for memory and attention deficits, as well as anxiety and depression-like phenotypes. Reversal of such cognitive and affective deficits by nicotine is even clearer for withdrawal-associated phenotypes. Tobacco smoking may thus also be maintained as a form of self-medication in individuals who show moderate cognitive or affective impairments and who are not diagnosed with a particular psychiatric condition. However, despite demonstrable nicotine-induced improvements of affective states and cognitive deficits, this is only indirect evidence supporting the self-medication hypothesis, which should not be considered as the only plausible explanation for high rates of smoking behavior in psychiatric populations. One should also emphasize the fact that chronic exposure to nicotine can also impair anxiety and mood in some conditions, to help attenuate hesitations in smoking-cessation attempts. Second, pre-existing phenotypes, such as high impulsivity and sensation seeking, appear to influence the appetence for nicotine according to most studies and may drive the propensity for initiating and pursuing smoking behavior. However, additional preclinical longitudinal studies need to be performed for resolving this issue, particularly to investigate the relationship between predisposing phenotypes and behavioral models that still need to be developed to truly capture addiction-like features such as habitual and compulsive nicotine taking and seeking. Last but not least, numerous studies reviewed here show that nicotine can trigger “pro-addiction” phenotypes such as impulsivity and deficits in cognitive flexibility. Nicotine-induced enhancements of learning, memory, and attention may also promote the shift toward nicotine addiction by facilitating the associations between smoking and contextual cues that underlie habitual drug use, craving, and relapse.

The great heterogeneity regarding the effects of nicotine observed across the different studies that we reviewed further suggests that the underlying reasons for smoking may vary across individuals, according to their pre-existing differences in genetics, life experiences, tobacco history, or personality traits.

## Author Contributions

All authors listed, have made substantial, direct and intellectual contribution to the work, and approved it for publication.

## Conflict of Interest Statement

The authors declare that the research was conducted in the absence of any commercial or financial relationships that could be construed as a potential conflict of interest.
